# Supplementary material for: Tissue-Specific Metabolic Reprogramming during Wound-Induced Organ Formation in Tomato Hypocotyl Explants
Source: Int J Mol Sci. 2021 Sep 18;22(18):10112. doi: 10.3390/ijms221810112 (PMC8466849; doi:10.3390/ijms221810112)
Supplement: Supplementary file 1 [file ijms-22-10112-s001.zip › 00_Larriba_ijms_supplemental.pdf]

Figure S1

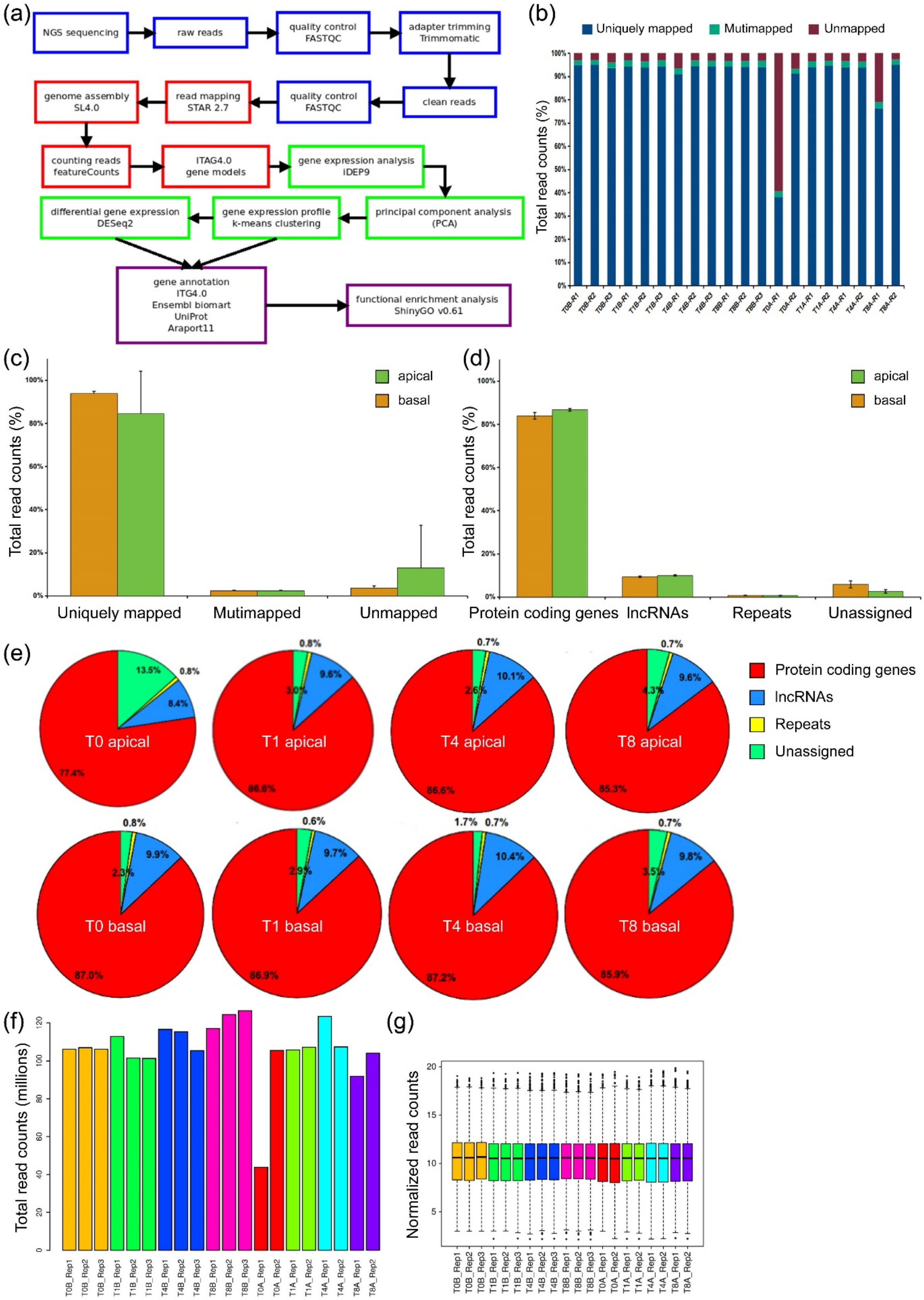

Figure S1. Bioinformatics pipeline, read mapping and gene expression statistics. (**a**) Bioinformatics workflow. Blue: cleaning and quality evaluation of Illumina sequencing libraries; red: mapping and gene counting; green: library normalization and differential

expression; purple: functional annotation and GO enrichment analyses; (**b**) Mapping results of the sequencing libraries; (**c**) Mapping result summary; (**d**) Summary of read counts distribution after genome features annotation; (**e**) Read counts distribution after genome features annotation of the different samples studied; (**f**) Number of reads assigned to protein coding genes in each sequencing library; (**g**) Box plot of reads assigned to protein coding genes in each sequencing library after normalization.

Figure S2

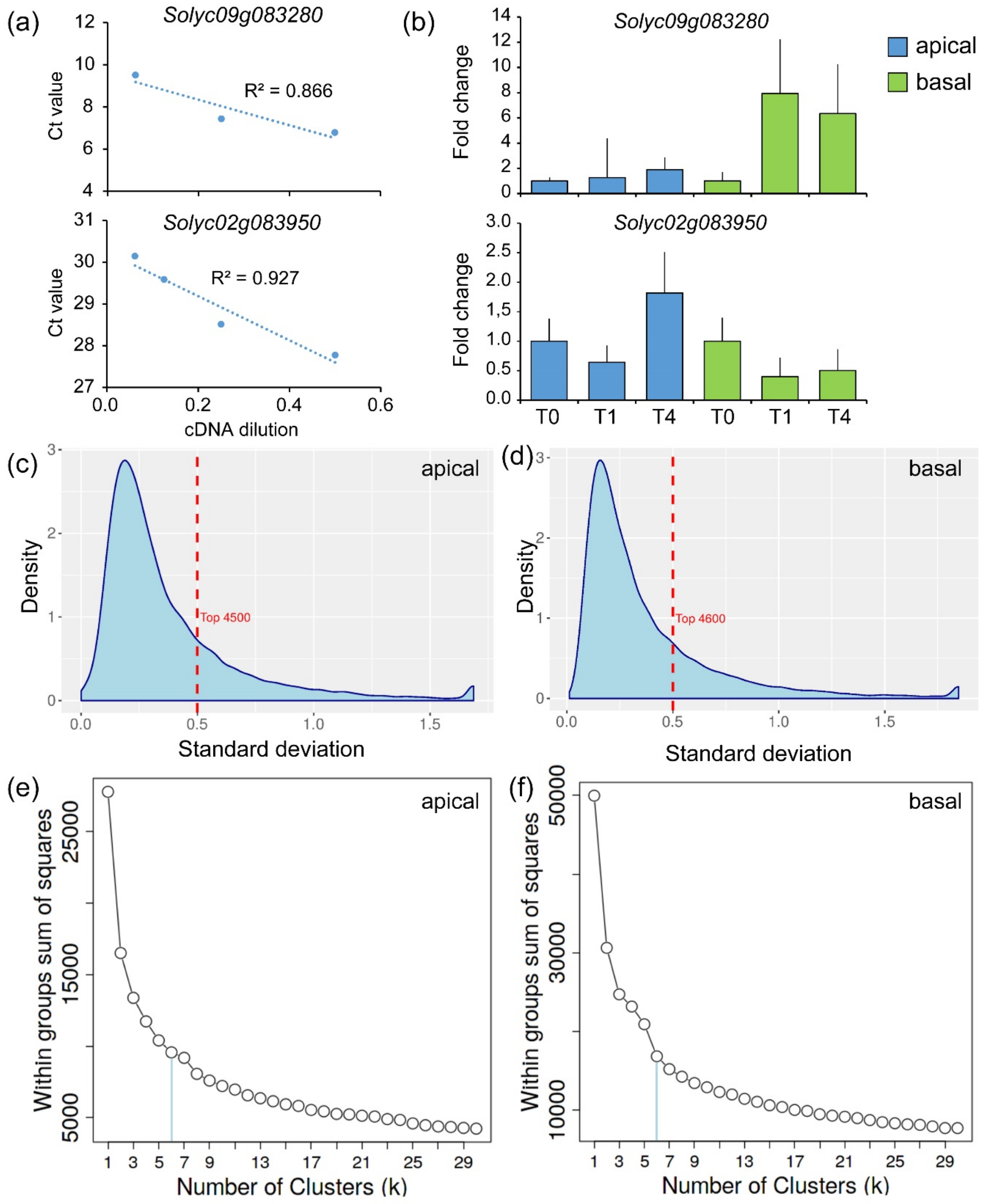

Figure S2. RNA-seq validation by RT-qPCR and K-means clustering analysis. (**a**) Primer validation of selected genes. Each dot represents the relative expression data for a given sample; (**b**) RT-qPCR of the expression of selected genes. Bars indicate normalized expression levels ± standard deviation (SD) relative to the hypocotyl explant at T0; (**c**, **d**) Distribution of gene expression SDs of the RNA-Seq results from apical (c) and basal (d) regions of the hypocotyl explants; (**e**, **f**) Identification of k-means optimal cluster number by Elbow analysis of the RNA-Seq results from apical (e) and basal (f) regions of the hypocotyl explants.

Figure S3

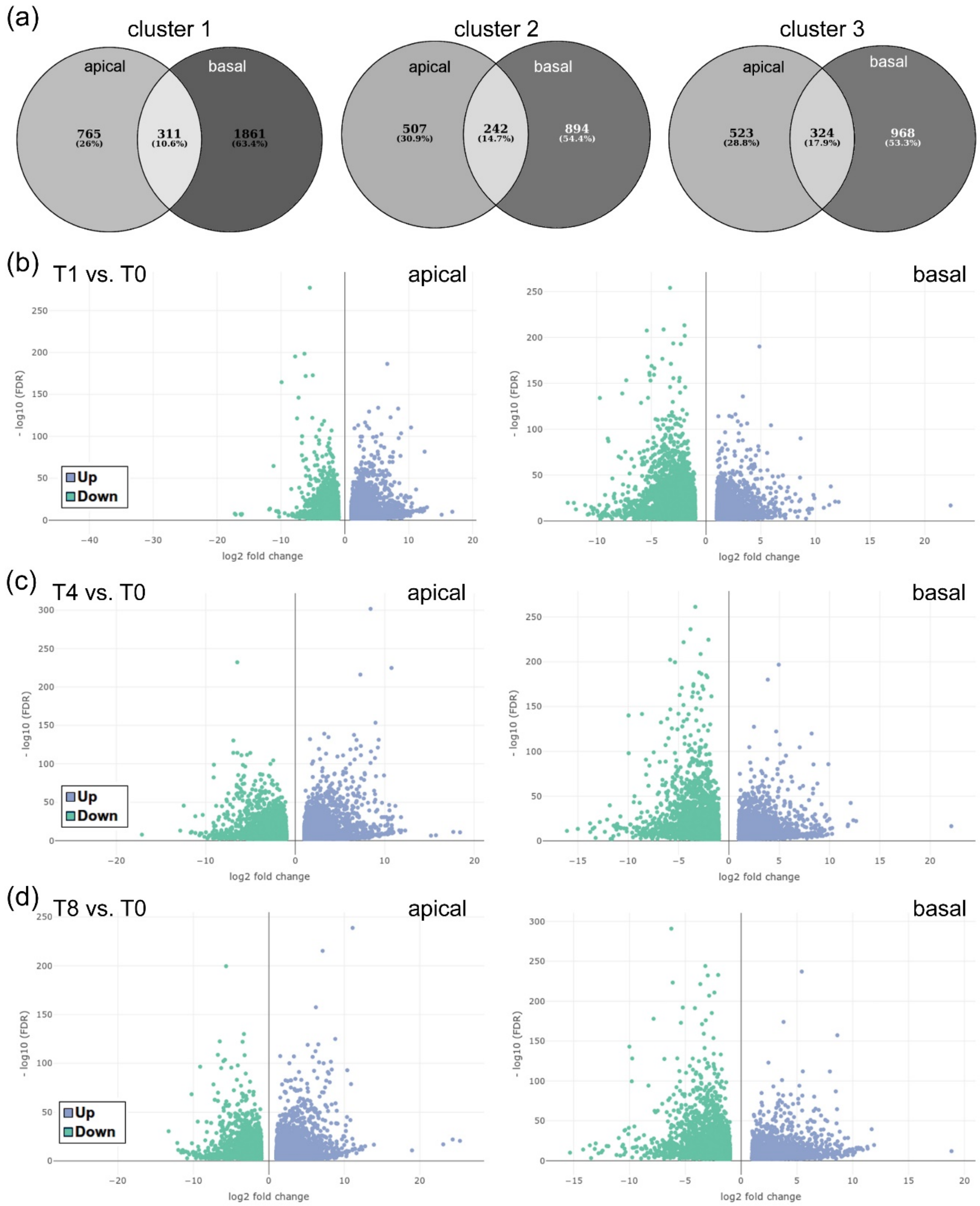

Figure S3. Venn diagrams and volcano plots of the RNA-seq results. (**a**) Venn diagrams from shared k-means clusters between the apical and basal regions of the hypocotyl explants; (**b-d**) Volcano plots of contrast between T1 and T0 (b), T4 and T0 (c) and T8 and T0 (d) from apical and basal regions of the hypocotyl explants. The log_2fold change is plotted on the x-axis, and the negative log_{10} (FDR) is plotted on the y-axis. The green/blue dots indicate the differentially expressed genes (DEG) with the absolute value of log_2fold change> < |1| and FDR<0.01.

Figure S4

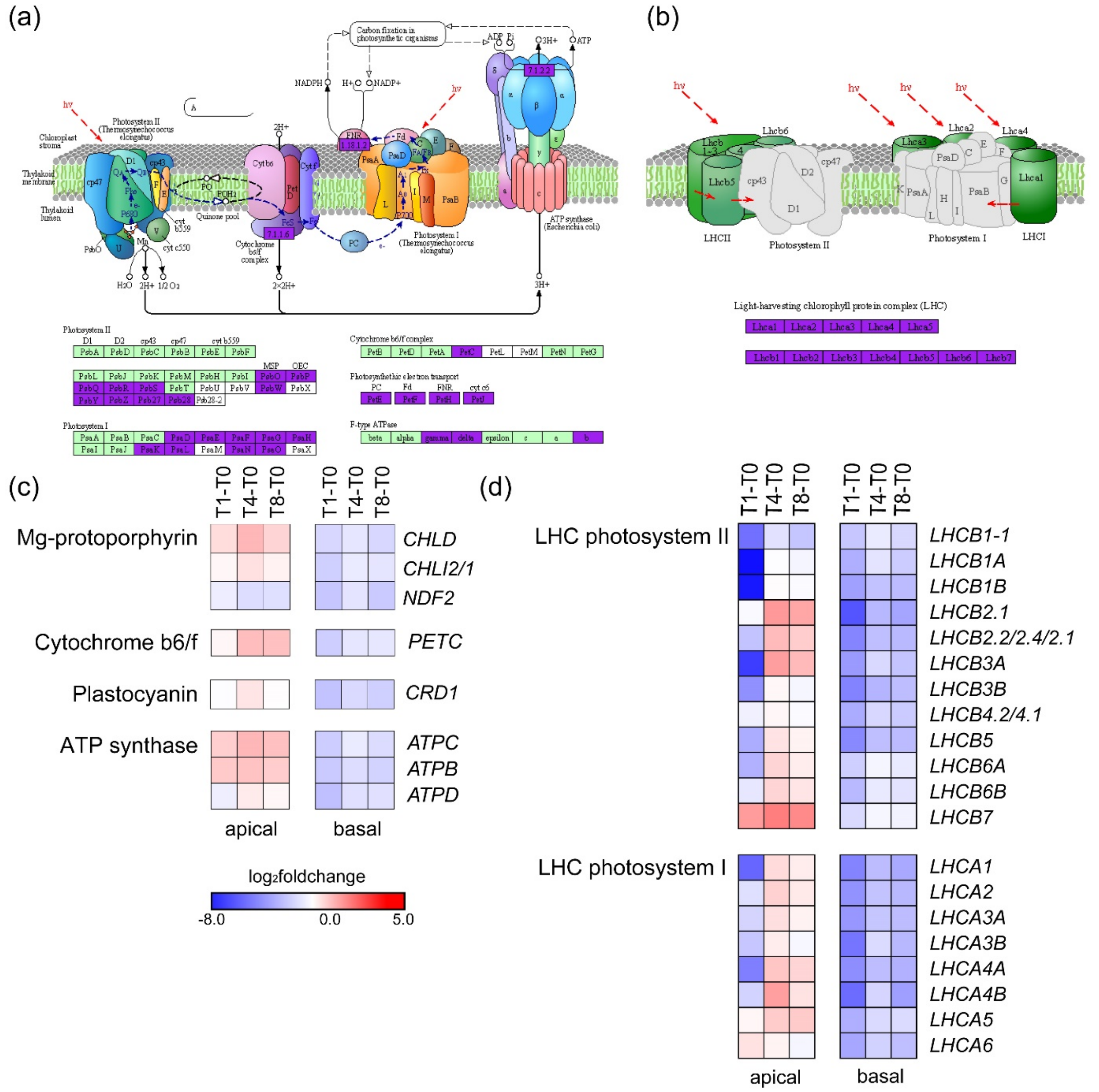

Figure S4. Expression of photosynthesis-related genes during wound-induced organ formation. (**a**) Photosynthesis pathway and (**b**) light-harvesting chlorophyll protein complexes (LHC) based on the Kyoto Encyclopedia of Genes and Genomes (KEGG) database (Kanehisa et al., 2021); *hv* designates light energy/photons. Genes expressed in our RNA-seq are indicated in light green, and those deregulated were indicated in purple; (**c**, **d**) DEG involved in know photosynthesis subcomplexes (c) or encoding the antenna proteins of the LHC (d). Gene annotations are found in Table S4.

Figure S5

(a)

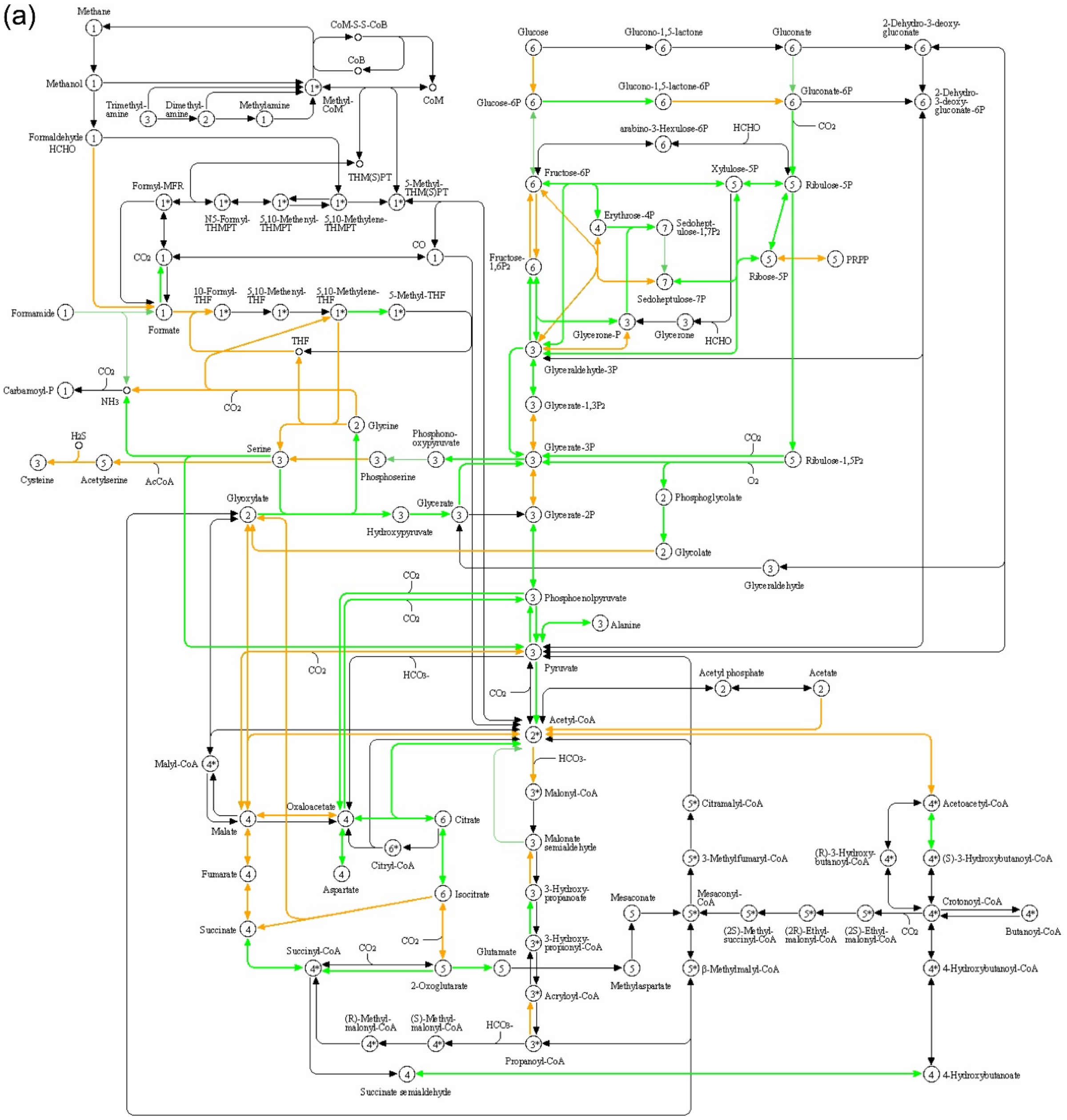
Methane
Methanol
Trimethyl-amine
Dimethyl-amine
Methylamine
Methyl-CoM
CoM-S-S-CoB
CoB
CoM
Formaldehyde HCHO
THM(S)PT
5-Methyl-THM(S)PT
Formyl-MFR
N5-Formyl-THMPT
5,10-Methenyl-THMPT
5,10-Methylene-THMPT
CO
CO2
Formamide
Formate
10-Formyl-THF
5,10-Methenyl-THF
5,10-Methylene-THF
5-Methyl-THF
THF
Carbamoyl-P
NH3
Glycine
Serine
Phosphoserine
Phosphono-oxypyruvate
H2S
Cysteine
Acetylserine
AcCoA
Glyoxylate
Hydroxypyruvate
Glycerate
Glucose
Glucono-1,5-lactone
Gluconate
2-Dehydro-3-deoxy-gluconate
Glucose-6P
Glucono-1,5-lactone-6P
Gluconate-6P
2-Dehydro-3-deoxy-gluconate-6P
arabino-3-Hexulose-6P
HCHO
Fructose-6P
Xylulose-5P
Ribulose-5P
Erythrose-4P
Sedohept-ulose-1,7P2
Fructose-1,6P2
Ribose-5P
PRPP
Sedoheptulose-7P
Glycerone-P
Glycerone
Glyceraldehyde-3P
Glycerate-1,3P2
Glycerate-3P
Ribulose-1,5P2
O2
Phosphoglycolate
Glycerate-2P
Glycolate
Glyceraldehyde
Phosphoenolpyruvate
Alanine
Pyruvate
HCO3-
Acetyl phosphate
Acetate
Acetyl-CoA
Malyl-CoA
Malonyl-CoA
Malonate semialdehyde
Oxaloacetate
Malate
Citrate
Citryl-CoA
Aspartate
Fumarate
Isocitrate
Succinate
Succinyl-CoA
2-Oxoglutarate
Glutamate
3-Hydroxy-propanoate
3-Hydroxy-propionyl-CoA
Acryloyl-CoA
Propanoyl-CoA
Mesaconate
Methylaspartate
Citramalyl-CoA
3-Methylfumaryl-CoA
Mesaconyl-CoA
β-Methylmalyl-CoA
(2S)-Methyl-succinyl-CoA
(2R)-Ethyl-malonyl-CoA
(2S)-Ethyl-malonyl-CoA
(R)-Methyl-malonyl-CoA
(S)-Methyl-malonyl-CoA
Acetoacetyl-CoA
(R)-3-Hydroxy-butanoyl-CoA
(S)-3-Hydroxybutanoyl-CoA
Crotonoyl-CoA
Butanoyl-CoA
4-Hydroxybutanoyl-CoA
4-Hydroxybutanoate
Succinate semialdehyde

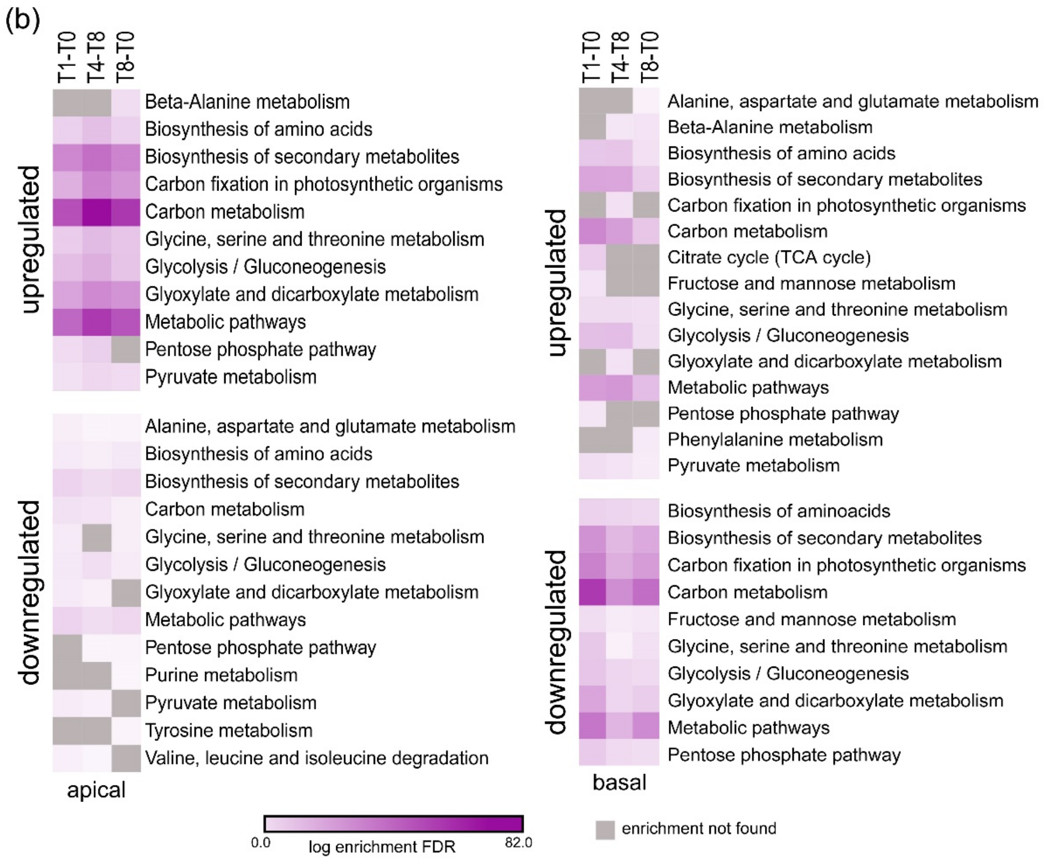

Figure S5. Deregulation of carbon metabolism genes during wound-induced organ formation. (**a**) Carbon metabolism pathway based on the KEGG database. Green and orange arrows respectively represent genes found deregulated in at least one of the contrast and genes expressed but not found deregulated in our RNA-seq; (**b**) Enrichment of specific carbon metabolism modules. Gene annotations are found in Table S5.

Figure S6

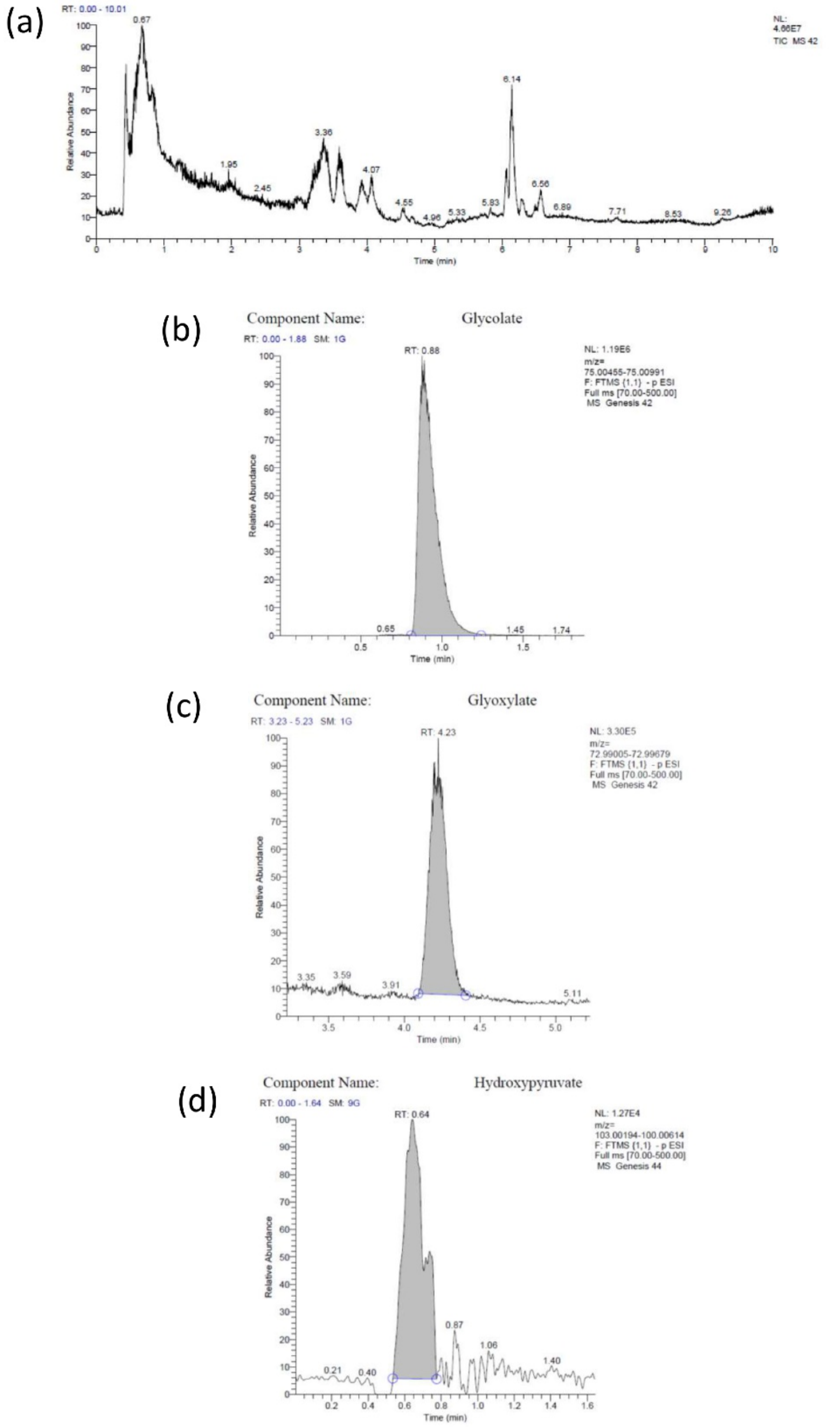

Figure S5. Supporting U-HPLC-HRMS chromatograms of metabolites of the photorespiration pathway in a selected hypocotyl sample. (**a**) Total ion chromatogram (TIC) at full scan obtained in the negative mode; (**b**, **c**, **d**) Target compound chromatograms of glycolate (b), glyoxylate (c), and hydroxypyruvate (d) obtained by extracting the exact mass from the TIC at a mass tolerance of ≤ 1 ppm.

Table S1. Primers for RT-qPCR validation of RNA-seq data. Gene name and gene identifier in ITAG4.0 annotation (Solyc ID) are included. Nucleotide sequence of the forward and reverse primers and amplicon size (cDNA) are indicated.

Table S2. Biological process (BP) GO term enrichment analysis from k-means gene clustering. List of BP GO terms found in enrichment analysis of specific and common genes found in k-means clustering. Common genes of apical and basal tissues, as well as the specific expression genes of both tissues, for clusters C1, C2 and C3 (see Figure 1) were indicated as: exclusive apical, exclusive basal and common, respectively. In addition, the enriched terms belonging to clusters 4 and 5, specific to the apical tissue, are shown as clusters 4 and 5, respectively. For each group of genes, the FDR value obtained in the enrichment analysis is indicated, the genes present in each analyzed group belonging to that term, as well as the number of genes noted in the background for that term. BP functional terms were sorted according to fold enrichment and FDR<0.01.

Table S3. Log2 fold change and FDR (adjusted p-values) values of DEGs during wound-induced organ formation.

Table S4. Biological process (BP) GO term enrichment analysis from DEG. In each Venn diagram subset, we compared upregulated and downregulated DEG in the apical and basal regions from three different contrast, T1-T0, T4-T0 and T8-T0. Functional categories are sorted according to fold enrichment and FDR<0.01.

Table S5. Annotation of photosynthesis-related genes in tomato. SolycID and ITAG4.0 annotation were retrieved from SolGenomics (https://solgenomics.net/). Putative *Arabidopsis thaliana* orthologs were identified from the Ensembl Plants database using BioMart. %id. Target A. thaliana, percentage of identity of target *Arabidopsis thaliana* gene identical to tomato gene; %id. tomato gene, percentage of identity of target tomato gene identical to *Arabidopsis thaliana* gene; confidence, orthology confidence score from the Emsembl Plants database. KEGG identifiers and annotations were retrieved using a strategy RBBH as described in M&M section.

Table S6. Annotation of carbon metabolism genes in tomato. See Table S5 legend for details.

Table S7. Transcription factor binding sites that were overrepresented in the promoters of up-regulated photorespiration genes.

Table S8. Annotation of genes encoding sugar transporters, invertases and sucrose synthases in tomato. See Table S5 legend for details.

Table S9. Growing media used in this work.
